# Hurricane Risk Variability along the Gulf of Mexico Coastline

**DOI:** 10.1371/journal.pone.0118196

**Published:** 2015-03-13

**Authors:** Jill C. Trepanier, Kelsey N. Ellis, Clay S. Tucker

**Affiliations:** 1 Department of Geography and Anthropology, Louisiana State University, Baton Rouge, Louisiana, United States of America; 2 Department of Geography, University of Tennessee, Knoxville, Tennessee, United States of America; University of California Los Angeles, UNITED STATES

## Abstract

Hurricane risk characteristics are examined across the U. S. Gulf of Mexico coastline using a hexagonal tessellation. Using an extreme value model, parameters are collected representing the rate or *λ* (frequency), the scale or *σ* (range), and the shape or *ξ* (intensity) of the extreme wind distribution. These latent parameters and the 30-year return level are visualized across the grid. The greatest 30-year return levels are located toward the center of the Gulf of Mexico, and for inland locations, along the borders of Louisiana, Mississippi, and Alabama. Using a geographically weighted regression model, the relationship of these parameters to sea surface temperature (SST) is found to assess sensitivity to change. It is shown that as SSTs increase near the coast, the frequency of hurricanes in these grids decrease significantly. This reinforces the importance of SST in areas of likely tropical cyclogenesis in determining the number of hurricanes near the coast, along with SSTs along the lifespan of the storm, rather than simply local SST. The range of hurricane wind speeds experienced near Florida is shown to increase with increasing SSTs (insignificant), suggesting that increased temperatures may allow hurricanes to maintain their strength as they pass over the Florida peninsula. The modifiable areal unit problem is assessed using multiple grid sizes. Moran’s *I* and the local statistic *G* are calculated to examine spatial autocorrelation in the parameters. This research opens up future questions regarding rapid intensification and decay close to the coast and the relationship to changing SSTs.

## Introduction

Hurricanes are tropical weather events capable of causing extreme destruction on land. Since the active North Atlantic season of 2005, research on the risk of hurricane events has been at the forefront of atmospheric disciplines. In just one season, 15 hurricanes formed, one being the now infamous Hurricane Katrina. Katrina caused at least $108 billion worth of property damage and was by far the costliest hurricane to ever strike the United States [1]. Though the event had a U. S. landfall in Louisiana, the damage was felt far beyond that. For example, storm surge penetrated more than 6 miles inland in coastal Louisiana, Mississippi, and Alabama, 42 tornadoes were reported in Georgia, Alabama, and Mississippi, and of the 1833 total fatalities, more than 250 occurred outside of Louisiana [2].

Across the Gulf of Mexico, hurricane wind risk varies greatly [3]. Risk, here, refers to the probability of an area experiencing a hurricane in a given time period and the likely intensity of the storm. It is separate from loss of life or livelihood. In the literature, much focus is on regional comparisons of risk [3–5] or attempting new methodologies to estimate risk [6–8]. Keim et al. [4] focus on 45 coastal locations from Texas to Maine and identify three regions of high activity. It is the goal of this research to add to the literature focused on localized (city-level) risk. The motivation for providing localized risk comes from several recent studies indicating risk variability along the Gulf of Mexico coastline. This is clearly illustrated in Keim et al. [4], especially in regard to the historical return rates of major hurricanes. Trepanier [3] shows that, in addition to the rate of hurricane occurrence, the range of hurricane wind speeds, and the maximum intensity for hurricanes also show great variability here. Ellis et al. [9] shows that extreme tropical cyclones in the Gulf of Mexico operate across a different temporal pattern than other regions and suggests that there are mechanisms causing variability within the Gulf Coast itself. Cohen [10] also proposes treating the Gulf Coast as separate hurricane regions. The goal of this study is to expand upon these ideas by providing a city-level analysis that details the variability of tropical cyclone risk across the Gulf of Mexico coastline.

Due to the rare nature of the most extreme hurricane events, it is difficult to model the expected occurrence or intensity using statistics. Estimating local risk exacerbates this problem because the amount of extreme data at such a small location is even more limited. One approach, known as extreme value theory (EVT), provides the theoretical framework that makes risk estimation of this type possible. Probabilistic EVT deals with the stochastic behavior of the maximum and the minimum of independent and identically distributed random variables. The distributional properties of extremes, as well as of exceedances over (below) high (low) thresholds, are determined by the upper and lower tails of the underlying distribution [11]. In particular, extreme value analysis usually requires estimation of the probability of events that are more extreme than have ever been observed [12]. EVT provides a framework that enables this type of extrapolation. This is a necessary extrapolation when using limited data sources or data with only a few extreme values.

Localized risk analysis is seen in the literature. Chu and Wang [13] focused on areas just outside of Hawaii, Malmstadt et al. [14] used EVT to estimate risk across 12 cities in Florida, and Scheitlin et al. [15] estimated risk at Eglin Air Force Base in northwestern Florida. At this point, however, a spatial analysis of local hurricane risk across the Gulf of Mexico coastline has not been done using EVT. Trepanier [3] estimated the regional risk of hurricane winds across the North Atlantic using the parameters from an EVT model. The size of each grid was roughly the size of Texas. The goal here is to use similar methodology for grids of much smaller scale and focus on the spatial variability across the U. S. Gulf of Mexico coastline.

Anyone who has driven across Interstate 10 in the southern United States knows that the surface topography and vegetation varies greatly from the west to the east. From the southern tip of Texas to the southern tip of Florida, the risk of extreme hurricane winds, varying winds, and the occurrence of winds is no different. The goal of this study is to visualize this spatial variation and then estimate the sensitivity of those different risks to sea surface temperature. The knowledge of this spatial variation and sensitivity to SSTs could be used to provide information for flooding models in a specific coastal location when a hurricane threat is imminent.

## Materials and Methods

### Extreme Value Model

The data used for this study include the National Hurricane’s Center’s (NHC) best-track data (HURDAT) and the Earth System Research Laboratory gridded sea surface temperature (SST) data. The R programming language [16] is used for all analyses and modeling.

The NHC best-track data set contains location and intensity estimates of observed tropical cyclones across the North Atlantic basin, including the Gulf of Mexico and Caribbean Sea. The history and origin of the data set are detailed in Jarvinen et al. [17]. The data set, also referred to as HURDAT, is maintained by the U. S. National Oceanic and Atmospheric Administration (NOAA) at the NHC. The version of the HURDAT file used in this study contains cyclones over the period 1854 through 2013 [18]. HURDAT provides information every six hours over the cyclone’s lifespan. For the purpose of spatial analysis and modeling, the six-hourly sampling resolution is at times too coarse. Therefore, we use a version of the data set that interpolates the six-hourly values into one-hour intervals. The advantage of the hourly interpolated values is that the chance of missing a hurricane passing through any given area is greatly reduced. The procedure is detailed in Elsner and Jagger [19].

The spatial analysis is done using equal-area hexagons that tessellate the northern Gulf of Mexico coastline, as seen in Elsner et al. [8] and employed in Trepanier [3]. In order to construct the hexagons, the set of hourly latitude and longitude locations for all cyclones of at least tropical storm intensity (≥ 18 ms^−1^) are projected onto a planar coordinate system. The rectangular area that contains all of the tropical storm locations is used as the study domain and is broken down into equal-area hexagons. The area of the hexagons is carefully selected so that the grids are large enough to have a sufficient number of tropical storm samples to model local risk, while being small enough that local variations are still meaningful. Here, an area of just under 23 thousand square kilometers is chosen (about the size of New Jersey) for the initial model run. This compares to an area of 629 thousand square kilometers that was used by Trepanier [3] to analyze basin-wide risk. The area used for this study is the smallest these grids can be made while still allowing each to have enough data for the model to successfully compute. Different hexagon sizes are used as an attempt to understand the influence of the modifiable areal unit problem in this study area.

**Fig 1:**
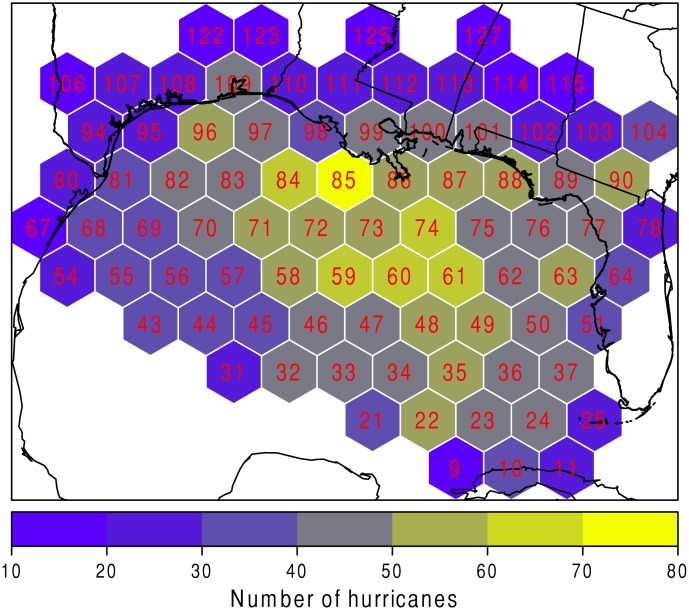
Number of hurricanes over the study area. The number of hurricanes per hexagon is shown using a color scale. The red number indicates the hexagon identification number.

The first step to analyzing regional risk is determining the highest individual hurricane intensity in each hexagon. Since a storm may spend several hours, and therefore have several data points, within one hexagon, the highest wind speed for each hurricane is found for each hexagon that they intersect. If a hexagon has fewer than 12 sample hurricanes then it is not included in the analysis. The procedure results in 83 hexagons. The number of hurricanes per hexagon are shown in Fig. 1. Hexagon identification numbers are also shown in red. The hexagon with the greatest sample size is hexagon 85 (just off the coast of Louisiana), having 73 maximum events recorded. The majority of maximum events are recorded in the center of the Gulf of Mexico, with a drop off in numbers after crossing the coast. The low numbers recorded at the southern edge of the study area are likely due to the way the data are subset. For example, an event’s track may have missed (skipped) the outer edge hexagon due to its forward speed.

The next step is to employ an extreme value model that provides a continuous estimate of a threshold intensity (return level) for a given frequency (return period). The model has previously been used in Jagger and Elsner [6], Malmstadt et al. [14], Trepanier [3], Trepanier and Scheitlin [20], and Ellis et al. [9]. As explained in these studies, a generalized Pareto distribution (GPD) describes the set of fastest winds above some high intensity threshold. The intensity threshold must be a compromise between having enough values to estimate the model parameters, but not too many that the intensities fail to be described by an extreme distribution.

As detailed in Jagger and Elsner [6], given a threshold wind speed *u*, the exceedances are modeled, *W* − *u*, as samples from a GPD family so that for an individual hurricane with maximum winds *W*, the probability that *W* exceeds any value *v* given that it is above the threshold *u* is given by

$$\begin{array}{ccc} \text{Pr}(W>v|W>u) & = & {\left(1+\frac{\xi }{\sigma }\left[v-u\right]\right)}^{-1/\xi }\\ & = & \text{GPD}(v-u|\sigma ,\xi ) \end{array}$$ (1)

where *σ* > 0 and *σ* + *ξ*(*v* − *u*) ≥ 0. The parameters *σ* and *ξ* are scale and shape parameters of the GPD, respectively.

The frequency of storms reaching at least threshold intensity follows a Poisson distribution with a rate, *λ*_*u*_, the threshold crossing rate. The number of hurricanes per year with winds exceeding the threshold rate (*v*) is a thinned Poisson process with mean *λ*_*v*_ = *λ*_*u*_ Pr(*W* > *v*∣*W* > *u*). This is the peaks-over-threshold method and the resulting model is completely characterized for a given threshold *u* by *σ*, or scale, *ξ*, and *λ*_*u*_ [3, 6, 14].

The return period for any *v* has an exponential distribution, since the number of storms exceeding any wind speed *v* is a Poisson process with mean *r*(*v*) = 1/*λ*_*v*_. As shown in Malmstadt et al. [14] and Trepanier [3], by substituting for *λ*_*v*_ in terms of both *λ*_*u*_ and the GPD parameters then solving for *v* as a function of *r* the corresponding return level for a given return period can be estimated as

$$\text{rl}\left(r\right)=u+\frac{\sigma }{\xi }\left[{\left(r\;\cdot \;\lambda_{u}\right)}^{\xi }-1\right].$$ (2)

For values of *ξ* less than 0, the model provides a limiting wind speed given by

$$\begin{array}{c} u+\frac{\sigma }{|\xi |} \end{array}$$ (3)

The limit is highest for large values of *σ* and small values of *ξ* [3]. A more complete description of the statistical theory supporting this model is given in Coles [12].

### Geographically Weighted Regression Model

In addition to the observed and interpolated tropical cyclone track data, this study makes use of the NOAA Extended Reconstructed Sea Surface Temperature (ERSST) V3b dataset to calculate sensitivity values [21]. These data are maintained by the NOAA Earth System Research Laboratory Physical Sciences Division. For each grid point, the average August-September-October value is taken from 1854–2013. This is the same SST timeframe used in a similar study by Elsner et al. [22] and encompasses the most active period of hurricane season. The SST data are transformed from latitude-longitude grids to a Lambert conformal conic (LCC) projection with secant latitudes of 30^∘^ and 60^∘^N and a projection center of 60^∘^ W longitude (the same projection used by the NHC for seasonal summary maps).

The temperature of the sea’s surface plays an important role in the genesis and intensification of hurricanes [23, 24]. According to the Maximum Potential Intensity (MPI) equation for tropical cyclones, rising SSTs will lead to an increased average power for hurricanes [25, 26]. Due to the other numerous variables that dictate how and when a hurricane will intensify (e.g., upper-level wind shear, depth of the thermocline), an individual event will rarely reach its MPI [27, 28]. Previous studies have shown that the relationship between SST and MPI is not perfect [29] and that the sensitivity of hurricane intensity to changes in SST varies across space [3, 30]. Here we examine this relationship at a smaller resolution along the Gulf Coast by using geographically weighted regression (GWR). A GWR model is chosen as it allows the relationship between the response (hurricane intensity) and the explanatory variable (SST) to vary across the study area [31, 32], identifying locations where the explanatory variable has more or less of an influence over the response.

With GWR the SST parameter is replaced by a vector of parameters (i.e., *λ*_*u*_, *σ*, *ξ*, or the return level estimates), one for each hexagon. The relationship between the response vector and the explanatory variables is expressed mathematically as

$$\begin{array}{c} y=X\beta \left(g\right)+\varepsilon \end{array}$$ (4)

where *g* is a vector of geographic locations, here the set of hexagons with different latent variables and

$$\begin{array}{c} \hat{\beta }\left(g\right)={\left(X^{T}WX\right)}^{-1}X^{T}Wy \end{array}$$ (5)

where *W* is a weights matrix given by

$$\begin{array}{c} W=\exp\left(\frac{-D^{2}}{h^{2}}\right) \end{array}$$ (6)

where *D* is a matrix of pairwise distances between the hexagons and *h* is the bandwidth. The elements of the weights matrix, *w*_*ij*_, are proportional to the influence hexagons *j* have on hexagons *i* in determining the relationship between *X* and *y*. Weights are determined by an inverse-distance function (kernel) so that values in nearby hexagons have greater influence on the local relationship compared with values in hexagons farther away. The bandwidth controls the amount of smoothing. It is chosen as a trade-off between variance and bias. A bandwidth too narrow (steep gradients on the kernel) results in large variations in the parameter estimates (large variance). A bandwidth too wide leads to a large bias as the parameter estimates are influenced by processes that do not represent the conditions locally. Here, an adaptive bandwidth is chosen that allows the estimates to vary depending on the location of the samples, similar to Trepanier [3].

## Results

### Localized Risk of Extreme Hurricane Winds

**Fig 2:**
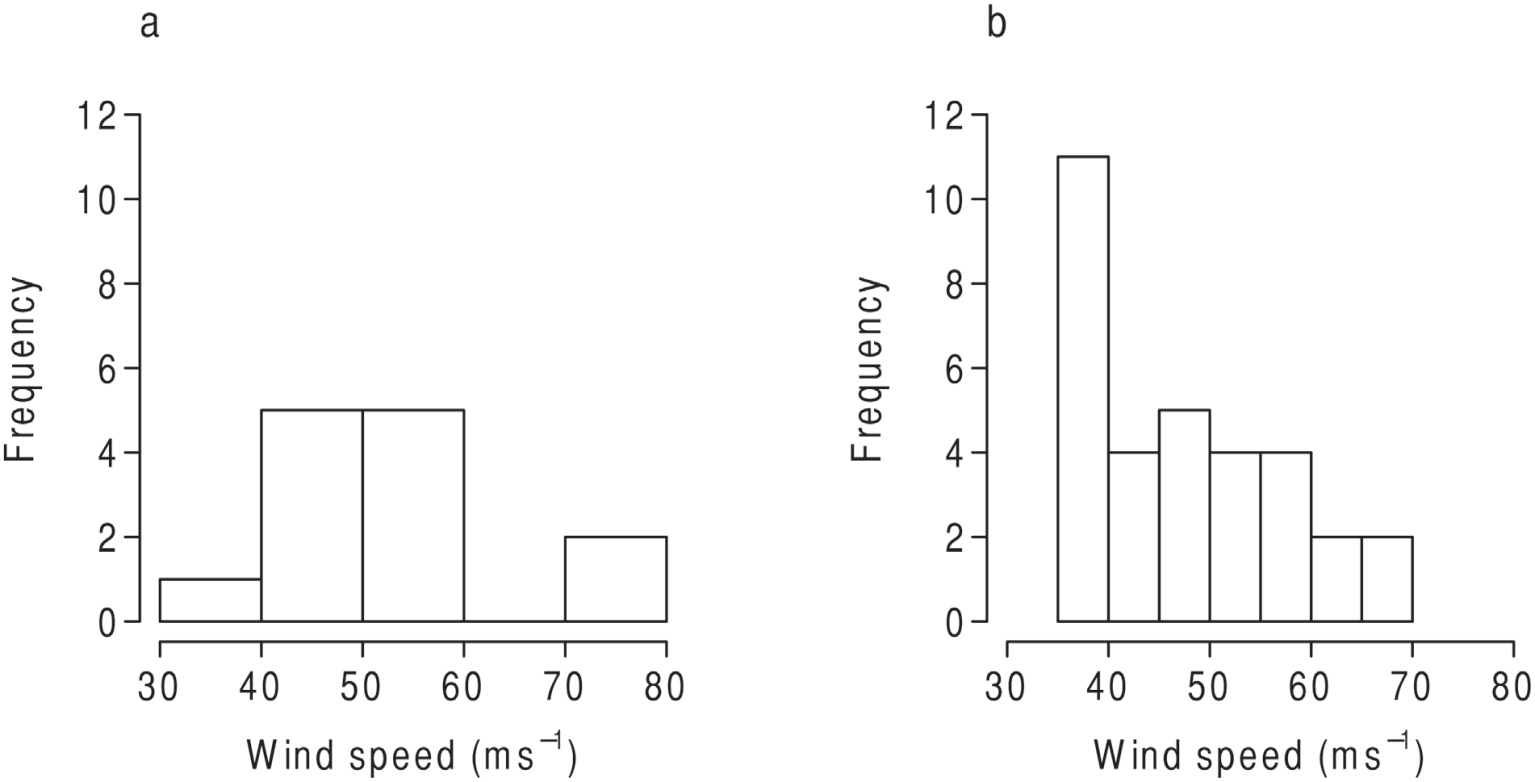
Histogram comparison of Grids 95 and 85. The maximum wind speed distributions are shown in ms^−1^ for Grid 95 (a) and Grid 85 (b).

**Fig 3:**
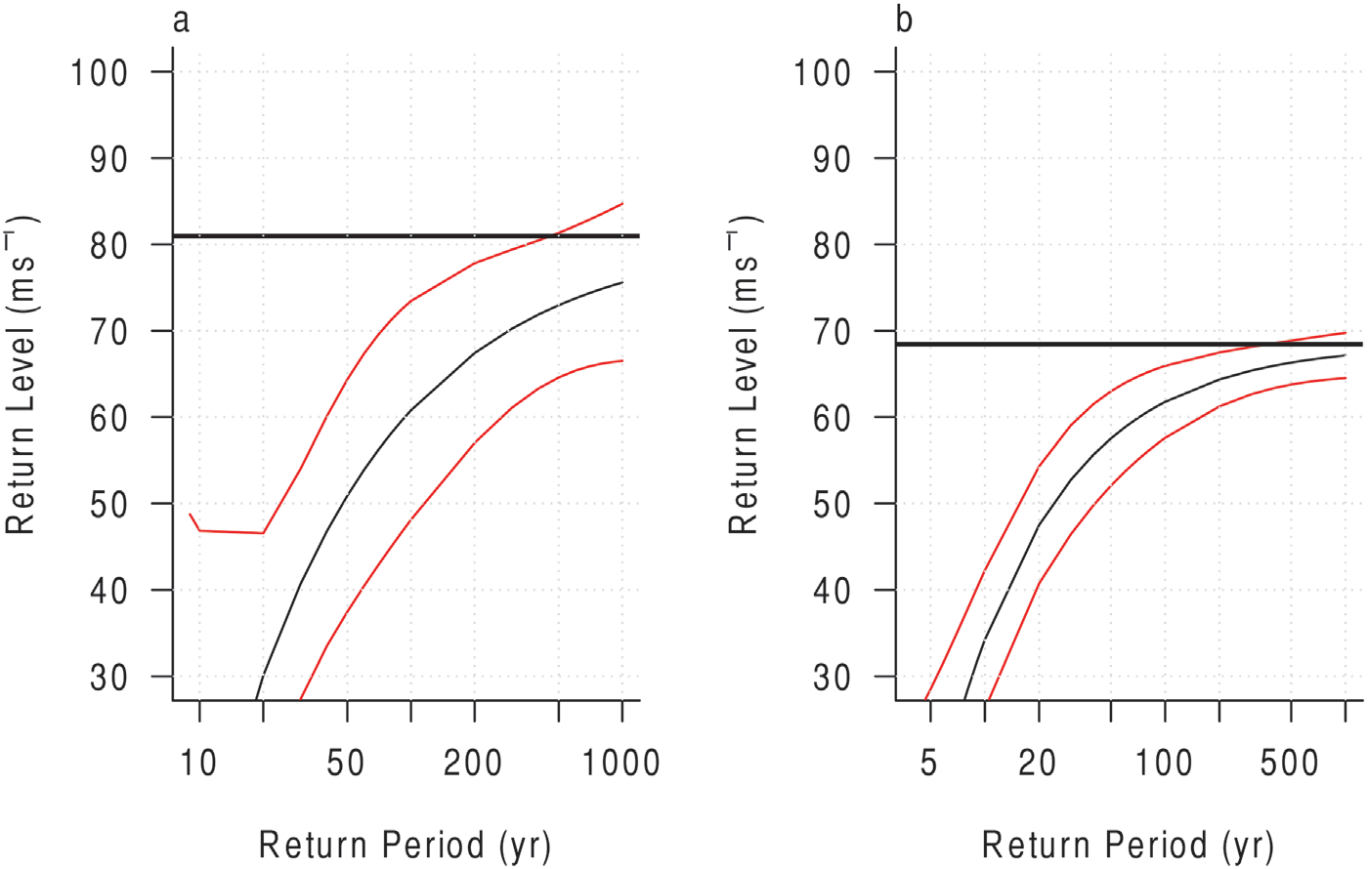
Return period curve for Grids 95 and 85. The extreme value model return period curves are shown for Grid 95 (a) and Grid 85 (b). The red lines indicate the 90% confidence bounds as calculated by the model. The horizontal, black line shows the theoretical maximum intensity for each grid.

The extreme value model is performed at all grids across the study area. Grids 85 and 95 are compared to illustrate the data that are used to estimate the model. Fig. 2 shows the histograms for grids 95 (Fig. 2a) and 85 (Fig. 2b). Although more maximum wind events were experienced in grid 85, the maximum value recorded in 95 is higher. The model uses the distribution information, such as the scale (dispersion) and shape (behavior of the tail), to calculate a threshold intensity for a given return period. Fig. 3 shows the calculated return level information for a range of return periods. Fig. 3a shows that the grid just to the east of central Texas has a 100-year return level of roughly 60 ms^−1^ (a low Category 4 event on the Saffir-Simpson scale). In comparison, Fig. 3b shows that the grid just to the south of central Louisiana has a 100-year return level just over 60 ms^−1^. The red lines indicate the 90% confidence bounds. The solid, horizontal black line indicates the theoretical maximum intensity. The model is calculated in the same fashion for each grid.

**Fig 4:**
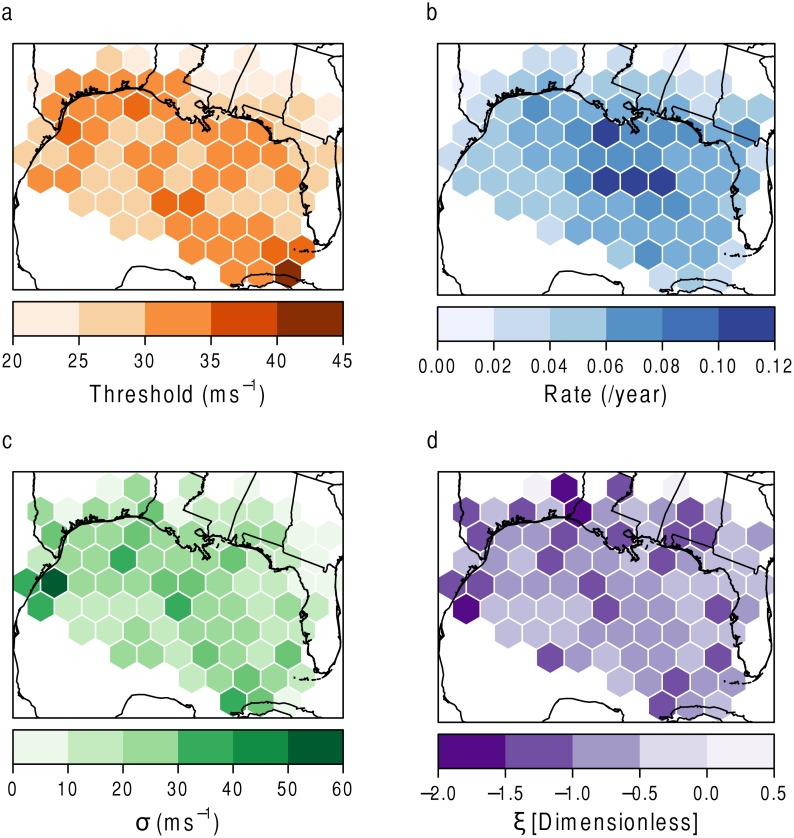
Extreme value model parameters mapped per hexagon. (a) Threshold (*u*) in ms^−1^, (b) Rate (*λ*_*u*_) of hurricanes per year, (c) Scale (*σ*) in ms^−1^, and (d) Shape (*ξ*).

Fig. 4 shows the variables of interest from the model. Fig. 4a shows the user-defined threshold parameter used in the model. It is taken as the 50*th* percentile of the distribution of wind speeds per grid. This is done so the results can be compared relative to one another [3] and to take into account that risk variables should be specific to the impacted location [9]. Fig. 4b shows the rate of occurrence per hexagon. The middle of the Gulf can expect to experience the most frequent events. Fig. 4c shows the *σ*, or scale, parameter. This parameter estimates the range, or dispersion, of wind speed values that can be expected. The darker the color, the wider the range of potential wind speeds. The southwestern corner, as well as the center, of the study area experience the events with the greatest variability in wind speed. Fig. 4d shows the *ξ*, or shape, parameter. This is a dimensionless parameter that indicates the tail behavior of the wind distribution. In other words, it represents the most extreme events. The closer the value is to −1, without going below, indicates a tail with the most severe extremes (the tail behavior drops off quickly). Below −1 and the model has problems fitting the distribution. There is not a definitive pattern in the *ξ* parameter. This is due to the sensitivity of the model to the most extreme events. One large value in a hexagon can cause the *ξ* parameter to vary greatly from nearby grids. This is looked at in more detail during the discussion of autocorrelation below.

**Fig 5:**
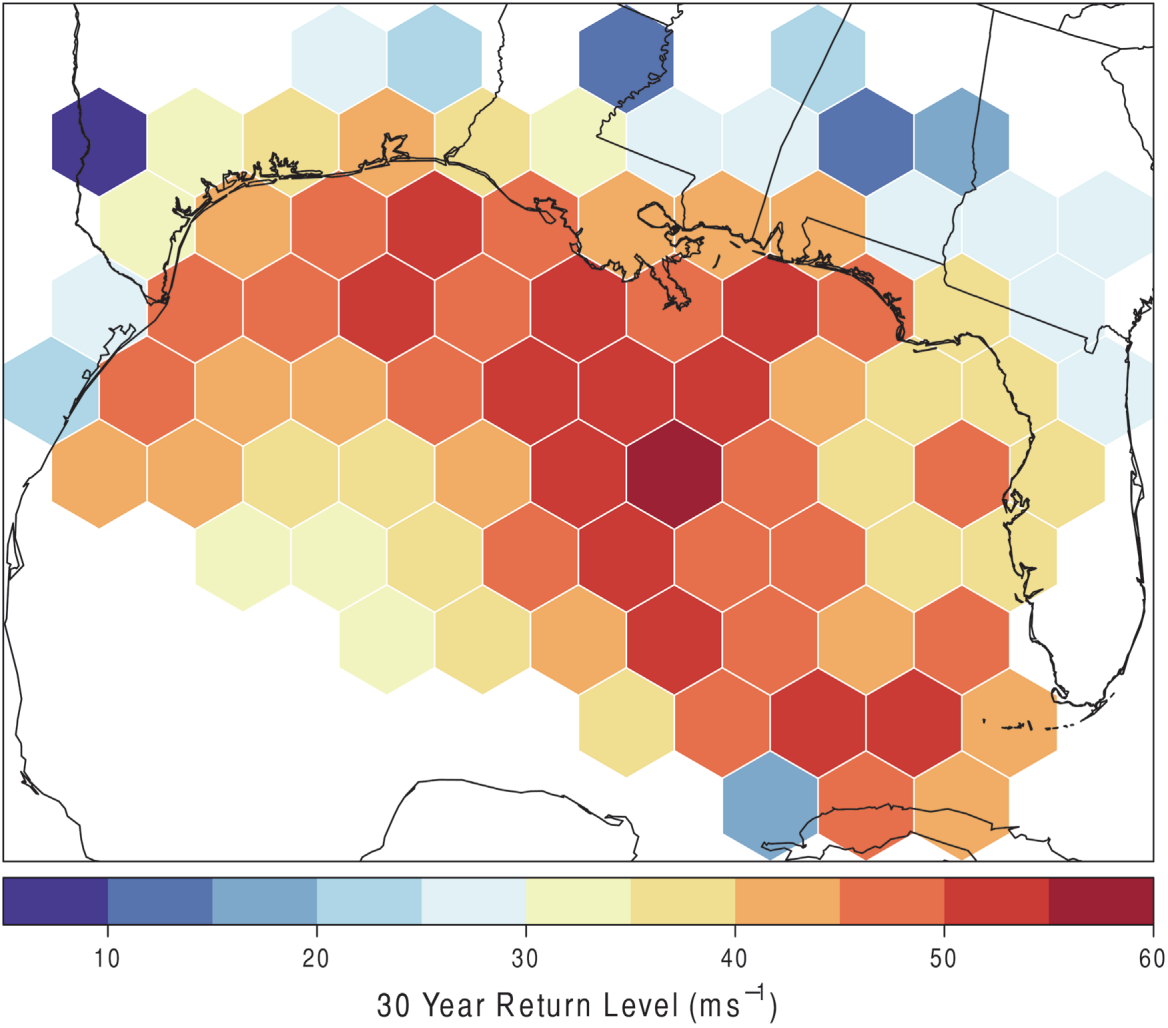
30-year return level in ms^−1^. The 30-year return level as output from the extreme value model mapped per hexagon.

Fig. 5 shows the 30-year return level per hexagon. Thirty years is chosen because that is enough time for all of the decadal cycles in the global climate to be accounted for, as well as to represent the typical homeowner’s mortgage [3]. This allows for the capturing of two important interests, the climate fluctuations and the broader public affected by hurricanes. The results can be interpreted as follows. Hexagon 85 can expect to experience a hurricane with wind speeds of at least 50 ms^−1^, on average, once every 30 years. It is important to note that the values mapped per hexagon exist somewhere in that grid. For example, just because a grid has a value of, say, 60 ms^−1^, it does not mean that everywhere in that grid will experience that wind speed. Rather, it suggests that at least one location within that grid will experience that intensity. The greatest 30-year return levels are mostly located in the center of the Gulf and along the coastline. Inland locations have a lower 30-year return level.

**Fig 6:**
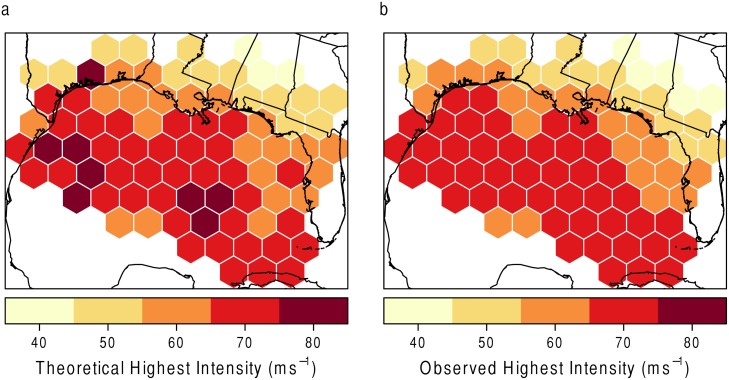
Comparison. (a) The theoretical maximum intensity in ms^−1^ per hexagon and (b) the observed maximum intensity ms^−1^.

As stated above, for values of *ξ* less than 0, the model provides a limiting wind speed. Fig. 6 shows a comparison between the theoretical highest wind as calculated by Equation 3 (Fig. 6a) and the observed highest wind as taken from HURDAT (Fig. 6b). This theoretical limit is calculated in a different way than the MPI equation given in Emanuel [33]. With the extreme value distribution limit, the theoretical highest intensity is calculated by using only the distribution of the observed data (*σ* and *ξ*) above a certain threshold *u*. The MPI equation takes into account the relationship between known hurricane characteristics and SSTs. As expected, none of the observed highest intensities surpass the theoretical. This suggests that the model is accurately depicting the environment from this perspective.

**Fig 7:**
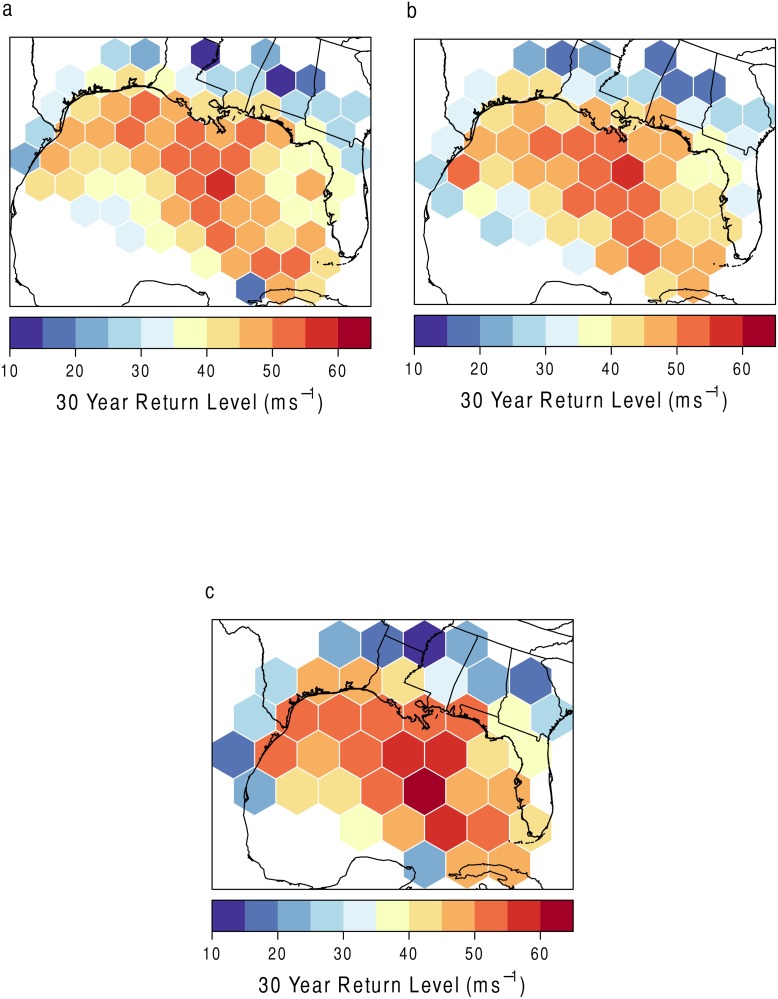
Test of the Modifiable Areal Unit Problem. The 30-year return level in ms^−1^mapped per hexagon at three different grid sizes. (a) Grid size used in the study. (b) and (c) are slightly larger. Grid (c) is used in the GWR model in the second portion of the analysis.

As an attempt to understand a potential modifiable areal unit problem (MAUP) and determine an appropriate hexagon size to understand local risk, different grid sizes are compared. MAUP refers to a source of statistical bias when various measures of spatial phenomena are aggregated [34]. Ideally, different regional aggregations should not alter the results. If they do, it suggests that the results are overly sensitive to the aggregation choice. Fig. 7 shows three different grid sizes with the 30-year return level as the variable of interest. Fig. 7a has grid sizes of roughly 23,000 km^2^ (the size used in the first portion of the analysis), Fig. 7b has grid sizes of roughly 30,000 km^2^, and Fig. 7c has grid sizes of roughly 61,000 km^2^ (the size used in the model created below). As shown in Fig. 7, the results stay fairly consistent even with changes in the grid sizes. In many grids, the 30-year return level increases slightly with an increase in grid size.

### Sensitivity to Sea Surface Temperature

**Fig 8:**
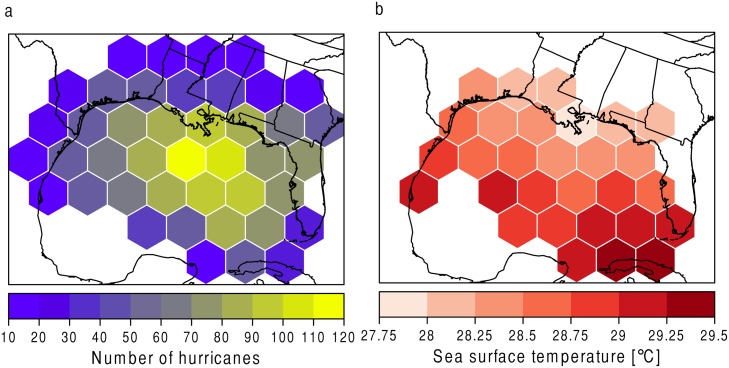
Number of hurricanes and SST over the study area. (a) The number of hurricanes per hexagon is shown using a color scale. (b) SST in degrees Celsius per hexagon.

The resolution of the SST data available for this study does not fit to the grid sizes used in the above portion of this paper. Therefore, the grids are made slightly larger to be able to incorporate the SST data. Fig. 8 shows the number of hurricanes (Fig. 8(a)) and the average August-September-October SST value (Fig. 8(b)) for each of the grid cells. As before, the extreme value model is run at each hexagon and the parameters and 30-year return levels are gathered.

To examine the influence of spatial autocorrelation in the parameters, two tests are conducted using the R package **spdep** [35]. Spatial dependence is an inherent property of an attribute in geographic space because of the continuity of space and the overall operation of various processes [36]. This dependence can be quantified to provide additional insight into the variability across space. The first is a global test of autocorrelation, known as Moran’s *I*. Moran’s *I* ranges between −1 and +1 with a value near 0 indicating no spatial correlation [37]. Moran’s *I* is defined as

$$\begin{array}{c} I=\frac{n}{s}\frac{y^{T}Wy}{y^{T}y} \end{array}$$ (7)

where *n* is the number of hexagons, *y* is the vector of values within each hexagon (e.g., rate parameter) where the values are deviations from the overall mean, *W* is a weights matrix, *s* is the sum over all the weights, and the subscript *T* indicates the transpose operator.

**Fig 9:**
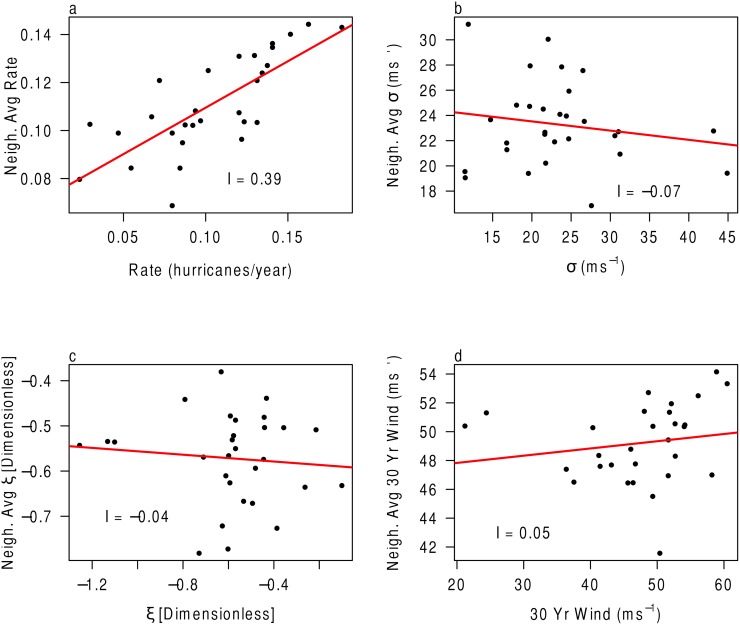
Test of spatial autocorrelation using the Moran’s *I* statistic. (a) Rate of hurricanes (per year). (b) *σ* in ms^−1^. (c) *ξ*. (d) 30-year return level in ms^−1^.

The spatial autocorrelation for each parameter can be seen in Fig. 9. For the *λ* parameter, Moran’s *I* is 0.39 and for the *σ* parameter it is −0.07. These values compare with −0.04 and 0.05 for the *ξ* parameter and 30-year return level, respectively. This suggests that the only parameter that is correlated across space is the *λ* or rate parameter. This makes sense since a hurricane occurring in one grid likely also occurs in a nearby grid as it completes its track.

The local statistic *G* is calculated for each grid [38] to explain local clustering of values. The basic statistic is defined as:

$$\begin{array}{c} G_{i}\left(d\right)=\frac{\sum_{j}w_{ij}\left(d\right)x_{j}}{\sum_{j}x_{j}} \end{array}$$ (8)

In this equation, the *x*_*j*_ are the weighted values of the points in the study area. *w*_*ij*_ is a binary, symmetric weights matrix with ones for all points *j* within distance *d* of point *i* and zeros otherwise. The difference between the local *G* statistic and Moran’s *I* is as follows. Moran’s *I* only tells you if your data are clustered, dispersed, or random. The *G* statistic tells the type of clustering, i.e., high vs. low. The *G* statistic measures the concentration of a parameter [38].

**Fig 10:**
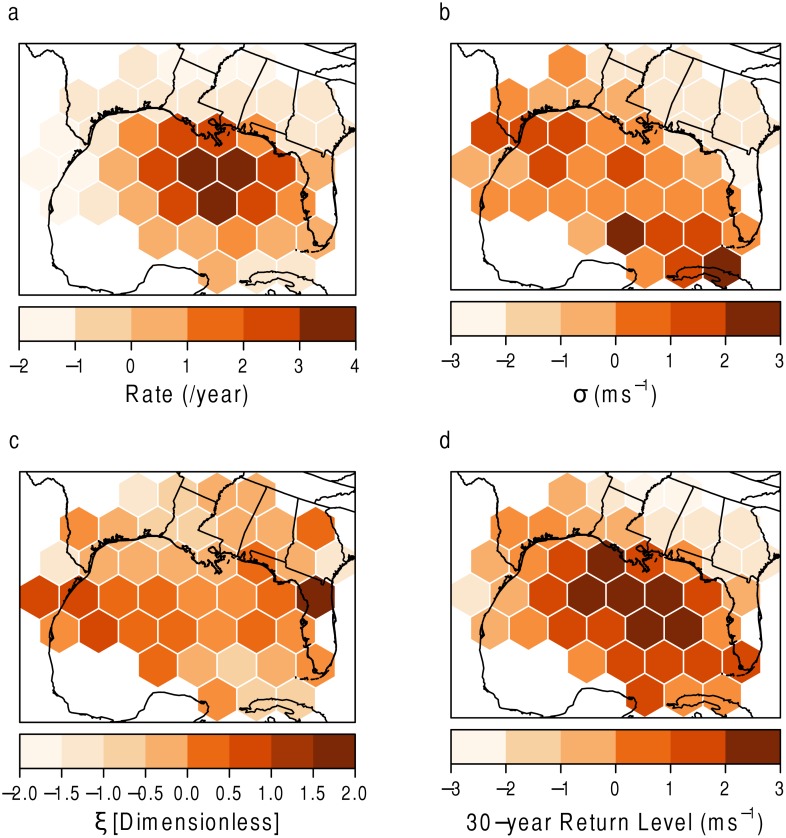
Test of spatial autocorrelation using the Local G statistic. (a) Rate of hurricanes (per year). (b) *σ* in ms^−1^. (c) *ξ*. (d) 30-year return level in ms^−1^.

The results are shown in Fig. 10. A positive (negative) value indicates a grid cell with a high (low) value surrounded by grid cells of high (low) values, or a local cluster of high (low) values. The Moran’s I indicated a clustering of the hurricane rate (*λ*), and the *G* statistic explains that the pattern consists of a cluster of high rates in the center of the Gulf and clusters of low rates inland (Fig. 10(a)). Similarly, there is a cluster of high 30-year return levels in the central portion of the study area (Fig. 10(d)).

The grids most sensitive to changes in SST are found using a geographically weighted regression (GWR) model. The percent change in the intercept values of each variable across the hexagons provides information showing how sensitive the latent variables are to SST. There is a −19.3% change in the *λ*, a 37.2% change in the *σ*, a 300% change in the *ξ*, and a −48.5% change in the thirty-year return level per degree SST. This suggests that the *ξ* parameter is the most sensitive to SSTs. The *ξ* parameter represents the most extreme events (negative values corresponding to more extreme events). These results are in opposition to those presented across the entire basin in Trepanier [3]. Trepanier [3] shows that the most extreme events (represented by *ξ*) become more extreme across the entire basin with increasing SSTs. Here, it is suggested that *ξ* becomes more positive (less extreme) with increasing SSTs this close to the coast. This is likely because there is not enough time for the storm to incorporate the energy provided by the warm SSTs into the system before the event makes landfall.

**Fig 11:**
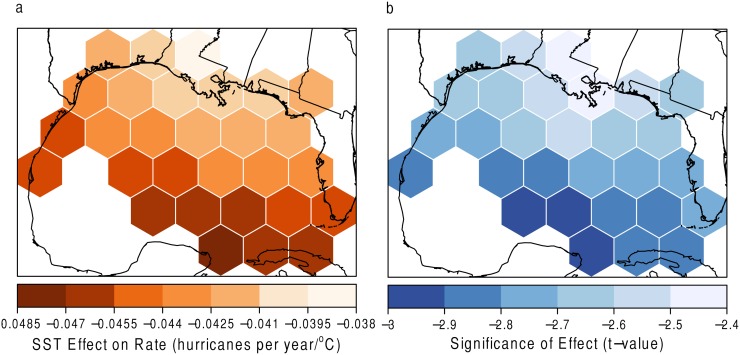
SST and Rate (*λ*). (a) The effect of SST on the *λ*_*u*_ parameter with (b) statistical significance. Grids with high *t* values (absolute value greater than 2) denote areas of statistical significance.

Local significance of the coefficients is found by dividing the SST coefficient by its standard error. The ratio, called the *t* value, has a *t*-distribution under the null hypothesis of a zero coefficient value. Grids with high *t* values (absolute value greater than 2) denote areas of statistical significance [3]. The results for the GWR model of SST on *λ* are shown in Fig. 11. The marginal influence of SST on the number of hurricanes per year is shown (Fig. 11(a)) along with corresponding *t* values (Fig. 11(b)). The negative relationship suggests that as SSTs increase, the frequency of hurricanes in these grids decreases. This relationship is significant in all grids (at the 5% level of significance). The negative relationship could be due to the close proximity to the coast. Few low pressure centers begin and turn into hurricanes in such a limited amount of space. Therefore, the number of hurricanes in grid cells close to the coast may be more related to SST in locations where tropical cyclogenesis is more prominent, as well as where the storm has traveled throughout its life span.

**Fig 12:**
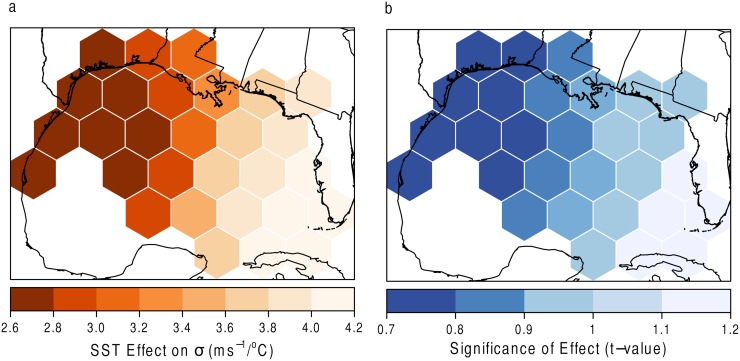
SST and *σ*. (a) The effect of SST on the scale parameter with (b) statistical significance. Grids with high *t* values (absolute value greater than 2) denote areas of statistical significance.

The results for the GWR model of SST on *σ* are shown in Fig. 12. In Fig. 12(a) it is shown that the scale of hurricane wind speeds in the eastern Gulf of Mexico, closest to Florida, are influenced by SST values more than in the other grids. This suggests that as SSTs increase, the range of wind speeds occurring in these hexagons will also increase. That is, more hurricanes of differing magnitudes will occur. The increased SSTs could allow for the possibility of hurricanes to maintain their strength as they pass over the Florida peninsula. The values are not statistically significant, however (at the 5% level).

**Fig 13:**
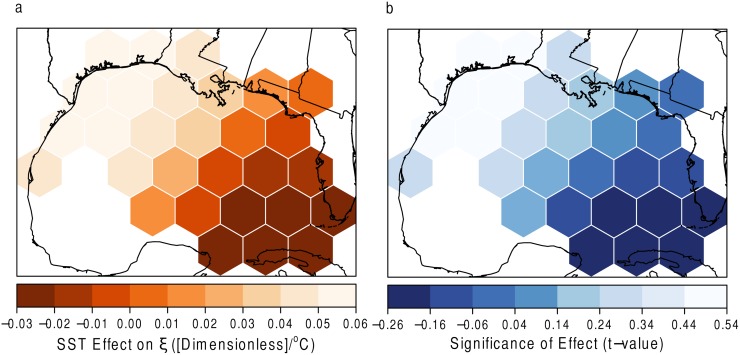
SST and *ξ*. (a) The effect of SST on the shape parameter with (b) statistical significance. Grids with high *t* values (absolute value greater than 2) denote areas of statistical significance.

The results for the GWR model of SST on *ξ* are shown in Fig. 13. In Fig. 13(a) it is shown that the parameter representing the shape of the wind speeds above the 50^*th*^ percentile (*u*) is influenced in opposite directions depending on the location. In the easternmost part of the Gulf (near Florida), the *ξ* parameter becomes more negative, suggesting those areas may experience more intense hurricanes with increasing SSTs. From the center of the Gulf westward, the values become more positive, suggesting that the events actually become less extreme with increasing SSTs. Although not significant (Fig. 13(b)), the geographic difference in the *ξ* parameter is interesting. Perhaps the warmer SSTs could, again, allow for events to maintain their intensities as they pass over the Florida peninsula. Whereas in the rest of the Gulf, the events are already nearing their maximum intensities and an increased SST plays very little to no influence.

**Fig 14:**
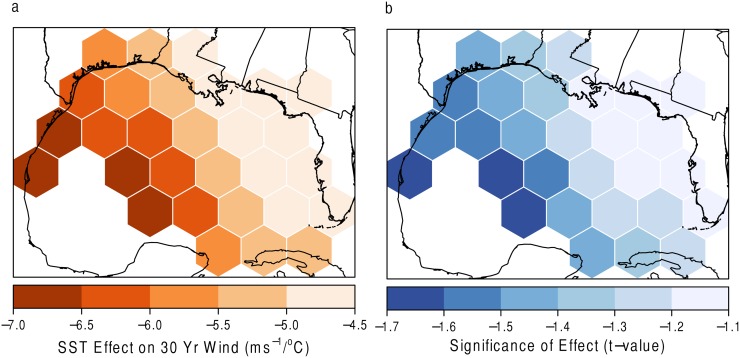
SST and 30-year return level. a) The effect of SST on the 30-year return level with (b) statistical significance. Grids with high *t* values (absolute value greater than 2) denote areas of statistical significance.

Finally, the results for the GWR model of SST on the thirty-year return level are shown in Fig. 14. In Fig. 14(a), SST values have the greatest influence at the western perimeter of the domain. Perhaps somewhat unexpectedly, as SSTs increase, the expected return level for a fixed time period decreases. These values are not statistically significant. This result is inconsistent with the results presented in Trepanier [3] for the entirety of the basin. Again, perhaps this close to the coast, the event does not have adequate time to gain strength off of an increased energy source.

Each of these plots suggests the overall influence of the ocean’s surface temperature on individual hurricane characteristics. Although three parameters were not significant, it provides an insight into hurricane characteristics that only a geographic approach can supply. It also supplies interesting evidence of the lack of importance of an increasing SST close to the coastline.

### Discussion

Hurricane characteristics vary significantly across space [3, 9]. Using a hexagonal tessellation, it is possible to visualize the local differences in these characteristics. Larger grid cell sizes can be used to assess large-scale variability [3, 30], while the smaller grid cells used here can better assess local risk. By incorporating a geographically weighted regression model, finding the sensitivity of these characteristics to changing sea surface temperatures is also possible. The focus of this study is on the northern Gulf of Mexico coastline. Not only does this region change significantly in topography from the east to the west, it also experiences a vastly different pattern of weather and climate. It is for these reasons it was hypothesized that the local characteristics of hurricanes would also change dramatically across the coast.

First, a grid roughly the size of New Jersey was used to examine the EVT model parameters and results. This is the smallest each grid can be while still allowing the model to run successfully (i.e., the model has enough data to fit the extreme value distribution). The results show that the parameters vary across space. *λ* is highest in the middle of the Gulf, *σ* is greatest south of Texas, *ξ* varies dramatically due to the role an extreme value can play on the model, and the 30-year return level is highest south of Louisiana, Alabama, and Mississippi. The influence of the modifiable areal unit problem was assessed and was found to be of little concern. Generally, as you increase the size of a grid cell the risk increases slightly.

In order to utilize SST data in a geographically weighted regression model, the grids were made slightly larger and the model was run again. Spatial autocorrelation was assessed at this point using a global and local metric. The only parameter significantly related to SST was the *λ*, or rate, parameter and in the opposite way one might expect. The model suggests that as SSTs increase, the frequency of hurricanes in those grids will decrease. It is hypothesized this is due to the close proximity to the coast and the unlikely chance tropical cyclogenesis will occur in any of these grids. Therefore, local SST may not be as important to determine hurricane frequency, and instead risk is determined by SST in locations of tropical cyclogenesis. The remaining parameters, though insignificant, showed interesting relationships with increasing SSTs. As one might expect, the range of expected wind speeds increases, particularly near western Florida. Perhaps events will have the necessary fuel with warmer waters to maintain their strength as they pass over the Florida peninsula. The *ξ* parameter varies across space, where again, Florida might be the determining factor. This close to the coast, it seems the expected hurricane intensity for a 30-year period is unaffected by increasing SSTs. This may be due to the lack of space that the event has to incorporate the increased fuel source.

The 30-year return level results in Fig. 5 were compared to the return periods found in Keim et al. [4]. The generalized Pareto distribution used in our study describes wind above a certain threshold and is theoretically more suited to estimate the most extreme wind speed values. The highest 30-year return levels along the coast in this study are along the Louisiana, Mississippi, Alabama, and Florida panhandle coastlines. The 30-year return *levels* in this study are compared to near-30-year return *periods* found in Keim et al. [4]. Keim et al. [4] has three sites along these coasts with near-30-year return periods for category 3 hurricanes (≥ 50 ms^−1^): Morgan City, LA (26 years), Boothville, LA (26 years), and Destin, FL (35 years). The results presented here show an average 30-year return level of (≥ 45–50 ms^−1^) for these locations, slightly more conservative than the 26-year return periods in Morgan City, LA and Boothville, LA.

## Concluding Remarks

Hurricane winds were analyzed for the U. S. Gulf of Mexico to determine the risk of high winds along the coastline. The frequency (*λ*, or rate) of hurricane-force winds is highest in the middle of the Gulf, decreasing toward the coast as friction from the land slows the winds. The widest range (*σ*, or scale) of wind speeds occurs just south of Texas. The 30-year return level is highest in the middle of the Gulf. However, this study analyzed wind risk along the coastline, specifically. The 30-year return levels along the coast are highest near the coastlines of Louisiana, Mississippi, Alabama, and the Florida panhandle. Additionally, these parameters were analyzed with respect to SSTs. A significant negative relationship exists between *λ* and SSTs. In other words, the frequency of high winds decreased as SSTs increased. This suggests that SSTs in likely places of tropical cyclogenesis, as well as along the storm’s track, are more important in determining frequency along the coast.

In a visual comparison of Fig. 5 in this study to Fig. 5 in Keim et al. [4], the highest risks are similar in both studies for the U. S. Gulf of Mexico coastline. The highest risk areas are Galveston, TX, southeast Louisiana, the coastline of Alabama, and the Florida panhandle. In contrast, the lowest risk areas are south Texas and western Florida.

This research opens up the opportunity for future questions. For example, intensification/decay rates could be analyzed at such a close scale to find if they are sensitive to SST change, since overall intensity is not. Near-shore SSTs likely play a larger role in the likelihood of rapid intensification and decay instead of overall hurricane frequency. Also, perhaps there is a particular hurricane track across Florida that is unique and is causing the parameters to vary in different ways in the eastern Gulf as compared to the west.

It is known that hurricanes vary across space. Using a tessellated grid at a local area allows one to see just how much these characteristics can change from one location to another over sometimes very short distances. These results can be used by local emergency managers to better prepare their specific locale for an event unique to their location.
